# Inclusion Body Myositis and Neoplasia: A Narrative Review

**DOI:** 10.3390/ijms23137358

**Published:** 2022-07-01

**Authors:** Laura Damian, Cristian Cezar Login, Carolina Solomon, Cristina Belizna, Svetlana Encica, Laura Urian, Ciprian Jurcut, Bogdan Stancu, Romana Vulturar

**Affiliations:** 1 Centre for Rare Autoimmune and Autoinflammatory Diseases (ERN-ReCONNET), Department of Rheumatology, Emergency Clinical County Hospital Cluj, 400347 Cluj-Napoca, Romania; ldamian.reumatologie@gmail.com; 2 CMI Reumatologie Dr. Damian, 6-8 Petru Maior St., 400002 Cluj-Napoca, Romania; 3 Department of Physiology, “Iuliu Hațieganu” University of Medicine and Pharmacy, 400006 Cluj-Napoca, Romania; 4 Radiology Department, “Iuliu Hațieganu” University of Medicine and Pharmacy, 400006 Cluj-Napoca, Romania; carolinasolomon12@gmail.com; 5 Radiology Department, Emergency Clinical County Hospital Cluj, 400006 Cluj-Napoca, Romania; 6 UMR CNRS 6015—INSERM U1083, University of Angers, 49100 Angers, France; cristina.belizna@wanadoo.fr; 7 Internal Medicine Department Clinique de l’Anjou, Angers and Vascular and Coagulation Department, University Hospital Angers, 49100 Angers, France; 8 Department of Pathology, “Niculae Stancioiu” Heart Institute Cluj-Napoca, 19-21 Calea Moților St., 400001 Cluj-Napoca, Romania; s_encica@yahoo.com; 9 Department of Hematology, “Iuliu Hațieganu” University of Medicine and Pharmacy, 400004 Cluj-Napoca, Romania; laura.urian@umfcluj.ro; 10 Department of Hematology, Ion Chiricuta Clinical Cancer Center, 400014 Cluj-Napoca, Romania; 11 Department of Internal Medicine, “Carol Davila” Central Military Emergency University Hospital, Calea Plevnei No 134, 010825 Bucharest, Romania; cjurcut@gmail.com; 12 2nd Surgical Department, “Iuliu Hațieganu” University of Medicine and Pharmacy, 400012 Cluj-Napoca, Romania; bstancu7@yahoo.com; 13 Department of Molecular Sciences, “Iuliu Hațieganu” University of Medicine and Pharmacy, 400349 Cluj-Napoca, Romania; romanavulturar@gmail.com; 14 Cognitive Neuroscience Laboratory, University “Babes-Bolyai” Cluj-Napoca, 400294 Cluj-Napoca, Romania

**Keywords:** inclusion body myositis, lymphocyte senescence, autophagy, mitochondria, cancer, interferon γ, large granular lymphocytes leukemia (LGLL), paraneoplastic, lymphocyte exhaustion, antibodies to the cytosolic 5′-nucleotidase 1A (anti-cN1A)

## Abstract

Inclusion body myositis (IBM) is an acquired, late-onset inflammatory myopathy, with both inflammatory and degenerative pathogenesis. Although idiopathic inflammatory myopathies may be associated with malignancies, IBM is generally not considered paraneoplastic. Many studies of malignancy in inflammatory myopathies did not include IBM patients. Indeed, IBM is often diagnosed only after around 5 years from onset, while paraneoplastic myositis is generally defined as the co-occurrence of malignancy and myopathy within 1 to 3 years of each other. Nevertheless, a significant association with large granular lymphocyte leukemia has been recently described in IBM, and there are reports of cancer-associated IBM. We review the pathogenic mechanisms supposed to be involved in IBM and outline the common mechanisms in IBM and malignancy, as well as the therapeutic perspectives. The terminally differentiated, CD8+ highly cytotoxic T cells expressing NK features are central in the pathogenesis of IBM and, paradoxically, play a role in some cancers as well. Interferon gamma plays a central role, mostly during the early stages of the disease. The secondary mitochondrial dysfunction, the autophagy and cell cycle dysregulation, and the crosstalk between metabolic and mitogenic pathways could be shared by IBM and cancer. There are intermingled subcellular mechanisms in IBM and neoplasia, and probably their co-existence is underestimated. The link between IBM and cancers deserves further interest, in order to search for efficient therapies in IBM and to improve muscle function, life quality, and survival in both diseases.

## 1. Introduction

Inflammatory idiopathic myopathies (IIM) are chronic multisystem diseases that may cause muscle, skin, and/or lung inflammation. Currently, IIMs are classified, based upon clinical, histopathological, and serological features, as dermatomyositis (DM), antisynthetase syndrome, immune-mediated necrotizing myopathy, inclusion body myositis (IBM), polymyositis (PM), and overlap myositis [1,2]. The most frequently acquired myopathy beyond middle age, IBM, is characterized by slowly progressive, asymmetrical muscle involvement, predominantly affecting the long finger flexors, quadriceps, and deglutition muscles [3]. The IBM pathogenesis involves autoimmune and degenerative mechanisms [4]. 

Typically, IBM is diagnosed upon the pattern of weakness, mildly elevated creatine kinase, and pathologic features. Classification criteria for IBM have been developed by the European Neuromuscular Centre [5]. Muscle biopsies reveal non-necrotic inflammation, mostly with T CD8+ lymphocytes, upregulation of major histocompatibility complex (MHC-I), abnormal protein aggregation in the sarcoplasm, and mitochondrial impairment [6]. The classically described rimmed vacuoles containing sarcoplasmic and myonuclear proteins, seen in 1–6% of cases, are no longer required for the diagnosis when the clinical picture is suggestive [3,7]. Electromyography reveals myopathic, neurological, or mixed patterns, while magnetic resonance imaging depicts the characteristic muscular involvement [8]. Additionally, antibodies to the cytosolic 5′-nucleotidase 1A (anti-cN1A or anti-NT51A/Mupp44) have been described in IBM [9]. 

The prevalence of IBM is 46/1 million, varying largely among countries and ethnic groups, likely reflecting disease recognition, or genetic differences [4,10,11]. The disease is 1.6 times (0.5–6.5) more frequent in males compared with females [4]. 

Adult-onset IIM has an increased risk of cancer, generally associated with DM and PM [12]. The overall rate of malignancies in 4538 patients with DM/PM was 12.1%, with a prevalence of malignancies occurring along with or after diagnosis of 14.8% for DM and 9.89% for PM, respectively [13]. In a recent large meta-analysis to estimate the malignancy risk in IIMs, standardized incidence ratios (SIR) of 4.66 for DM and 1.75 for PM have been reported [13]. It is possible for DM to precede a tumor by 3 years, but an increased malignancy risk persists even after 5 years [13,14]. 

There have been attempts to explain the association of myositis with cancer, as follows: cross-reactivity between the anti-tumor response and the regenerating muscles, tumor DNA somatic mutation, the inner activation of the checkpoint inhibitors pathway to increase the anti-tumor response, the tumor-infiltrating lymphocytes, and other mechanisms involving the immune surveillance [15,16]. 

## 2. Is IBM a Paraneoplastic Myositis?

The association between IBM and neoplasia is controversial [17]. Classically, IBM was not considered to be associated with an increased cancer risk. In long-term IBM studies the mortality was either not influenced [18,19], or increased, with a SIR of 1.7, but not this was not related to malignancies [12]. The most frequent causes of death were aspiration pneumonia and cachexia, while cancer was less commonly encountered than in general population [11,18,19]. The cancer risk was not increased in a recent Norwegian IBM cohort (SIR 0.9) [12]. In a study based upon the South Australian myositis database (in 133 patients followed up for 8 years) the association of IBM with cancer was also not significant, (SIR 1.37, 95% CI 0.76–2.27 as malignancies occurred in 11.3%, i.e., 15 cases, with respect to almost 11 expected cases) [20]. 

Nevertheless, a population-based cohort study found an increased risk of malignancy of IBM, SIR 2.4, (1.2–4.9) [21]. Additionally, a Brazilian series revealed 4 cases of cancer in 30 IBM patients [22]. In the large biopsy-proven series from Victoria, Australia, the SIR for malignancy in IBM was 1.8 (95% CI = 5.01–5.22) [20]. Cancer, mainly hematologic, was found to be amongst the most important IBM co-morbidities [23]. In a population-based case-control study derived from the Rochester Epidemiology Project, patients with IBM (*n* = 50) were 3.9 more likely to have a hematologic neoplasia [23].

An explanation for this discrepancy could be that paraneoplastic myositis is generally defined as the co-occurrence of malignancy and myopathy within 1 to 3 years of each other [12,14,15]. However, the onset of IBM is generally late, the symptoms are very slowly progressive, and the disease is often diagnosed only after 5 years of evolution [19]. Additionally, many studies of malignancy in IIM did not include IBM patients [20].

There are nevertheless reports of IBM in association with cancer, such as bladder carcinoma [24,25], renal cancer, uterine carcinoma [26], papillary thyroid carcinoma [27], and hematologic malignancies, including a series of large granular lymphocyte leukemia (LGLL) [28]. Furthermore, IBM was also reported in monoclonal gammopathy [29]. The most common cancers in a Norwegian IBM series were prostate, colorectal, and hematological malignancies [12]. 

The patients with IBM in the Rochester Epidemiology Project, besides the hematologic neoplasia proneness, were also 6.2 times more likely to have Sjogren’s syndrome (SjS) than population controls [23]. The prevalence of cancers in SjS is significantly increased, and mainly consists of hematological neoplasia including MALT (mucosa-associated lymphoid tissue) and other types of B-cell lymphomas, but can also consist of solid cancers [30]. 

## 3. Genetic Susceptibility for IBM and Tumors 

Sporadic IBM is associated with HLA-D3 and the 8.1 ancestral MHC haplotype, with haplotypic allelic combinations at the HLA-DRB1 (mainly DRB1*03:01) and HLA-B*-08:01 [31]. Furthermore, HLA DRB1*03 was also found in T-LGLL (T cell large granular lymphocyte leukemia), less common in responders to therapy [32]. Elevated HLA DRA expression was recently described in IBM [33], and HLA-DRA is a prognostic immune predictor in bladder cancer [34]. In IBM, the chemokine receptor CCR5 gene variants have also been found [4,33]. The C-C chemokine ligand 5/C-C chemokine receptor type 5 (CCL5/CCR5) axis is important in tumor progression, in hematologic as well as in solid tumors [35]. Additionally, a MYH2 gene variant increased the IBM risk in Japanese patients [36], and MYH2 was involved in colorectal carcinogenesis [37]. 

## 4. Inflammation in IBM, LGL and Other Cancers 

### 4.1. CD8+CD28 Null CD57+ Lymphocytes 

Greenberg et al. noted in 2016 that as many as 58% of IBM patients, screened prospectively, have aberrant clonal populations of T large granular lymphocytes (LGL), as in T-LGLL [28]. In IBM-associated T-LGLL, the clonal hyperplastic CD8+CD57+ T cell populations, prevalent in the invasive infiltrate in the endomysium and myofibers, were present in blood as well [28]. Similar associations of T-LGL clonal expansions have been observed in rheumatoid arthritis (RA) and SjS [38]. Not all clonal T-LGL expansions are true leukemia, as asymptomatic patients with normal blood counts may be classified as having T cell clonopathy of undetermined significance [32]. Additionally, the distinction of T-LGLL from reactive large granular lymphocytosis, such as viral infections, is difficult, as a skewed T cell lymphocyte repertoire with oligoclonal expansion is common to both [39].

The T-LGLL is a rare late-onset clonal disorder of CD8+ cytotoxic T cells, expressing CD57 [28]. The T-LGLL disorder is characterized by defective apoptosis of cytotoxic CD8+ T cells, due to the dysregulation of apoptotic pathways and the activation of survival signaling pathways [39,40]. Chronic activation of the Janus kinase–signal transducer and activator of transcription (JAK/STAT) pathway is a hallmark of T-LGLL [40]. Furthermore, an increased IFNγ-mediated signaling is found, related to a lack of negative regulators, namely the suppressors of cytokine signaling, SOCS1 and SOCS3 [40]. 

Evidence is accumulating in favor of the autoimmune etiology of IBM, which seems to be a clonal highly differentiated cytotoxic CD8+ T cell-mediated disease [4,6]. T regulatory cells (Tregs) and macrophages are also involved [33]. Bioinformatics analysis in IBM has recently identified hub genes, biological pathways, and processes, such as apoptosis, inflammatory response including the chemokine signaling pathway, IFN (mainly gamma IFN) response, IL2-STAT5 signaling, and the p53 pathways [33]. 

The CD8+CD28 null T cells found in IBM and T-LGLL are potent producers of IFNγ, both in muscle and in circulation [4,41,42]. These T cells are apoptosis-resistant, with reduced Fas expression and increased antiapoptotic molecule expression [43,44]. The CD8+CD28 null T cells also express TNFα, granzyme B, perforin, and killer cell lectin-like receptor G1 (KLRG1) [43,44]. The CD57 positivity and the CD28 co-stimulatory molecule loss are associated with lymphocyte senescence, a terminal differentiated state [28,43,44]. Moreover, the glucocorticoid resistance of these CD28 null T cells therapy is likely due to the loss of the glucocorticoid receptors [43]. Cytotoxic T cells in IBM also show signs of exhaustion, with decreased functional and proliferation responses, as the result of persistent antigen exposure and the expression of immune checkpoint PD-1 (programmed cell death protein-1) [44]. The senescence- and exhaustion-related signaling pathways are non-overlapping in the control of survival and replication [45]. 

Both senescence and exhaustion are dysfunctional states of cytotoxic T cells (Table 1). Senescence, a cellular stress response, is common in aging-related degenerative pathologies and malignancy [46]. Cells undergo senescence in response to oncogenic stimuli, tumor suppressive defects, genetic or epigenetic damage, repeated antigenic challenge, and a number of other causes [46]. Senescent cells, besides arrested growth (related to p53/21 and p16/RB pathway activation), secrete proinflammatory cytokines, chemokines, growth factors, and proteases belonging to the senescence-associated secretory phenotype (SASP) [46]. The SASP modulates angiogenesis, cell proliferation, inflammation. and tissue repair, but also modulates chemotherapy resistance in tumor niches [46,47]. Senescent CD8+ T cells, although non-proliferative, are reprogrammed to perform NK-like functions by upregulating genes for innate signaling adaptors, such as TYROBP, chemokines, and effector cytotoxic molecules [33,48,49]. The terminally differentiated CD8+ T cells express MHC class I and respond to IL-15 and IL-7 survival signals, regulated by CCR7 expression [47,50]. Additionally, IL15 is also a critical cytokine in T-LGLL [51].

T cell exhaustion occurs in cancers, as PD-1 inhibits immune responses and modulates T cell activity to promote tolerance [52]. Therefore, inhibition of the immune checkpoints PD-1, its ligand PD-L1, and the cytotoxic T-lymphocyte associated protein CTLA4 is an efficient antitumor strategy [52]. The PD-1/PD-L1 axis plays an important role in the immune system, along with the PI3k/AKT/mTOR pathway [52]. Additionally, PD-L1 expression is regulated by the JAK/STAT axis in cancer [52]. The tumor infiltrating lymphocytes may behave differently in cancers and myositis, either taking part in the muscle inflammatory infiltrate, or increasing the expression of checkpoint inhibitors in cancer by means of IFNγ [15].

### 4.2. Interferons in Inclusion Body Myositis and Cancer

Interferons (IFNs) are pleiotropic cytokines with antiviral, immunomodulatory and antitumor properties [53]. Type I (IFNα and IFNβ) and type II interferons (IFNγ) were detected in studies of IBM muscle biopsies [54,55,56]. In normal cells, IFNγ is under the control of the suppressor of cytokine signaling-1 (SOCS1) [40].

The IBM disorder is associated with a prominent IFNγ signature [54,55]. A recent analysis of the differentially expressed genes in IBM revealed enrichment of IFN signaling, along with other genes involved in Th1, Th2, macrophages, fibroblasts, and endothelial cells activation [57]. Moreover, IFNγ is most highly expressed early on in the disease course [54].

**Figure 1:**
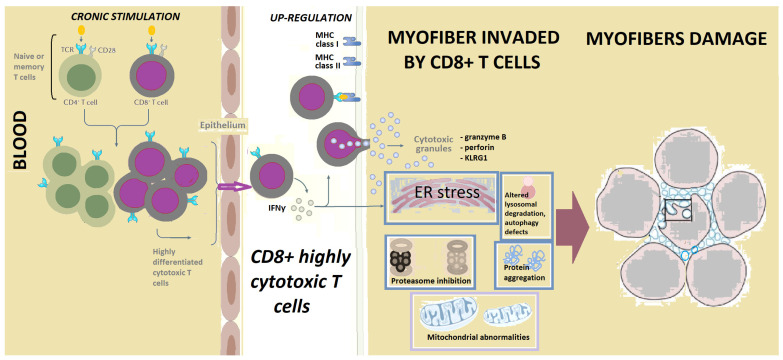
The terminally differentiated, CD8+ highly cytotoxic T cells, expressing NK features, are central in the pathogenesis of IBM. Antigenic stimulation of T cells results in the transformation of early effector memory T cells into a highly differentiated population of cytotoxic CD8+ T cells, present in inclusion body myositis blood and muscle. These highly cytotoxic T cells have escaped the requirements for co-stimulation (such as the loss of CD28), and they invade myofibers and produce cytotoxic granules. There are also issues including mitochondrial abnormalities, endoplasmic reticulum (ER) stress, autophagy defects, aggregation of proteins, and reduced mitochondrial ATP production. These highly differentiated CD8+ T cells have similar features to those present in large granular lymphocytic leukemia. Circulating T cells might explain autoimmune features in some patients with IBM.

Furthermore, IFNγ gene expression is increased in the myofibers invaded by CD8+ T cells (Figure 1), along with the IFNγ-inducible Th1 chemokines CXCL-9 and CXCL10, and other IFN-I related proteins [42,58,59].

The IFNγ produced by the cytotoxic CD8+ T cells results in upregulation of MHC class I and MHC class II in myofibers, through the class II MHC transactivator (CIITA) activation [4,55]. As a result, IFNγ causes endoplasmic reticulum (ER) stress and aggregation of proteins, including p62 and the hn-ribonucleoprotein TDP-43 [4]. Furthermore, IFNγ may impair myofiber repair [4].

The IFNγ synthesis is enhanced in an inflammatory or tumoral milieu by positive feedback [53]. The IFNγ activates the JAK/STAT pathways, including in cancer cells, with consecutive transcription of PD-L1 and other inhibitory ligands [60]. In cancers IFNγ mainly has antitumor effects, with cytostatic, pro-apoptotic, antiproliferative, and antiangiogenetic functions, and can induce Treg apoptosis [53].

Prolonged exposure of tumor cells to IFNγ is crucial for an antitumoral effect [53]. Nevertheless, low-dose IFNγ may favor tumorigenesis and metastasis via the downregulation of MHC and checkpoint inhibitors, such as PD-1, and by impairing cytotoxic T cell responses [53,61]. In lung, prostate, renal, breast, endometrial, and pancreatic cancer cells, low doses of IFNγ in the tumor microenvironment, generated by host immune cells or during cytokine therapy, may in fact favor tumor progression [53]. The appropriate dose of IFNγ in a tumor setting is currently unknown [53]. Furthermore, IFNγ levels are elevated in T-LGLL [40].

### 4.3. Stat Transcription Factors in IBM and Cancers/ LGLL 

Some signaling pathways (Table 2) in IBM have also been described in tumorigenesis [33]. The transcription factors of the JAK/STAT family, secreted upon IFN stimulation, are involved in IBM, and in cancers as well, including T-LGL [4,53].

A transcription factor responding to inflammatory signals, STAT1, was found in IBM muscle biopsies proteomics [40,56,59,62]. Elevated phosphorylated STAT1 (pSTAT-1) has also been described in T-GLL, along with elevated levels of IFNγ [40]. Dysregulation of the IFNγ–STAT1 signaling pathway in T-LGLL likely results from low levels of IFNγ receptor and from the decreased suppression of cytokine signaling-1 (SOCS1) [40].

Another transcription factor, STAT3, intervenes in cell proliferation, survival, differentiation, angiogenesis, and myogenesis [63]. STAT3 downregulates the mechanistic target of rapamycin (mTOR) involved in muscle differentiation [64]. Physiologically, a transient STAT3 muscle expression is beneficial for muscle regeneration and hypertrophy; nevertheless, prolonged STAT3 exposure activates the caspase and ubiquitin proteasome system in the muscle, contributing to muscle wasting [64,65,66]. Therefore, STAT3 inhibition helps preserve muscle mass in cancer cachexia [65,66].

The constitutive activation of STAT3 is the hallmark of LGLL [28,39]. Additionally, STAT3 is activated in the majority of human cancers, as well as being involved in inflammation-induced tumor initiation and progression in cancers, such as those of the breast, brain, prostate, ovary, colon, and others [62,63]. In hepatocellular carcinomas, constitutive activation of STAT3 stimulates cell proliferation and tumor angiogenesis, decreasing apoptosis and antitumor immunity [64].

Generally, STAT1 and STAT3 are considered to have opposite effects, with STAT1 being considered a tumor suppressor and STAT3 an oncogene [62]. Nevertheless, STAT1 may promote tumorigenesis by sustaining inflammation [62]. Additionally, pSTAT1 is surprisingly correlated with STAT3 in some cancer cell lines including breast cancer and T-LGLL [40,62]. Furthermore, STAT3 upregulates STAT1 expression in cancer cells [62]. 

Dysregulated STAT5 signaling was recently described in IBM [33] and was also found in T-LGLL [67].

**Table 2:** Common pathogenesis in IBM and cancers/T-LGLL.

Common Pathogenic Mechanisms	In IBM	In Cancers/T-LGL	References
Genetic susceptibility	DRB1*03 is associated with IBM. HLA DRA is elevated in IBM. *CCR5* gene variants are found in IBM. A *MYH2* gene variant increases IBM risk in Japanese	DRB1*03 is associated with T-LGLL. HLA DRA is elevated in bladder cancer. CCL5/CCR5 axis is involved in hematologic and solid tumor progression. MYH2 is involved in colorectal carcinogenesis.	[4,31,32,33,34,35,36,37,68]
CD8+CD28− cytotoxic T cells	In IBM these cytotoxic T cells are clonally expanded and produce IFNγ.	In T-LGLL these cytotoxic T cells are clonally expanded and produce IFNγ.	[4,39,44,69,70]
Interferon γ	IBM is associated with a prominent IFNγ signature, mostly early during the disease.	IFNγ is elevated in LGL. IFNγ is crucial for antitumoral effects, but low-dose IFNγ may favor tumorigenesis by impairing cytotoxic T cell responses.	[4,53,54,55,61]
STAT1, STAT3	STAT1 in IBM muscle biopsies is elevated. STAT3 is involved in myogenesis.	pSTAT-1 in T-LGLL is elevated. STAT1, a tumor suppressor, may also promote tumorigenesis by sustaining inflammation. STAT3 constitutive activation is the hallmark of LGLL. STAT3 is involved in tumoral cachexia.	[4,39,40,53,56,59,62,63]
Anti-cN1A antibodies	In IBM anti-cN1A are associated with reduced muscle cN1A expression, mitochondrial abnormalities and myofiber intracytoplasmic protein aggregation of p62/SQSTM1. cN1A knockdown activates AMPK, which upregulates the muscle-specific ubiquitin ligases with muscle wasting.	Serum cN1A activity is decreased in breast cancer (possibly through inactivating antibodies), correlated with muscle damage parameters.	[7,64,71,72,73,74,75,76,77]
Mitochondrial abnormalities	In IBM mitochondrial size, dynamics, and function defects are progressive. SIRT1, regulating mitochondrial function, is low in IBM muscle, despite increased SIRT-1 mRNA. GDF15, a mitochondrial disease marker, is increased in IBM.	Mitochondria may favor cancer cells survival in oncogenesis. SIRT1 directly influences tumor progression, metastasis, and other oncogenic mechanisms. GDF15 is increased in cancers.	[6,78,79,80,81,82,83,84]
Autophagy	Sarcoplasmic aggregates of autophagy-associated proteins p62/SQSTM1, LC3 and TDP-43, involved in UPR and ER stress, are pathologic hallmarks of IBM. FYCO-1 missense variants are found in IBM vacuoles.	p62, a tumor suppressor, may accumulate in cancers due to autophagy defects. LC3, associated with autophagosome formation, is a marker of poor tumor differentiation. TDP-43 may function as tumor promoter or suppressor. FYCO-1 is associated with invasiveness and metastatic potential.	[59,73,85,86,87,88,89,90,91]
Chaperones	TCP-1 is overrepresented in IBM vacuoles.	Chaperonin-containing TCP-1 promotes tumor progression, chemoresistence and metastasis.	[85,92]
Ubiquitin-proteasome system	UPS dysfunctions are involved in IBM and in cancer. The ubiqutinase Atrogin-1/MAFbx is increased in IBM.	UPS dysfunctions are involved in cancer. Atrogin-1/MAFbx is increased in tumor cachexia.	[82,93,94,95]
Cell cycle	In IBM cell cycle markers Ki67, PCNA, cyclins D1, E are increased.	Ki67 and cyclins D1 and E are overexpressed in tumors.	[96,97,98]
MicroRNAs	MiR-133 is reduced in IBM.	MiR-133, a tumor suppressor, is reduced in acute myeloid leukemia and in other cancers.	[57,78,99]
Metabolic	In IBM metabolic profiles of activated cytotoxic CD8+T cells rely upon mitochondrial fatty acid oxidation.	Metabolic profiles of cancer cell and activated cytotoxic CD8+T cells are similar, relying upon mitochondrial fatty acid oxidation for survival.	[70]
Calcium homeostasis	In IBM Ca^2+^ homeostasis dysfunction is involved in the defective cytotoxic T cells apoptosis and mitochondrial defects. IBM may be a “functional calpainopathy”.	Aberrant calpain activation negatively impacts cancer prognosis.	[100,101,102]

Legend: AMPK: AMP—activated protein kinase; anti-cN1A—antibodies anti-cytosolic 5′nucleotidase 1A; ATG7—the autophagy gene 7; Atrogin-1/MAFbx—ubiquitin ligase muscle atrophy F-box; CCL5/CCR5—C-C chemokine ligand 5/C-C chemokine receptor type 5; ER stress—endoplasmic reticulum stress; FYCO1—FYVE and coiled-coil domain-containing protein-1; GDF15—growth differentiation factor-15; SSP—60-heapt shock protein-60; IFNγ—interferon gamma; miRNAs—microRNAs; pSTAT-1—phosphorylated STAT1; SIRT1—sirtuin-1; *SQSTM1*—sequestosome 1 gene; TCP-1—T-complex protein-1; TDP-43—TAR-DNA-binding protein-43; UPR—unfolded protein response; UPS—ubiquitin-proteasome system.

### 4.4. Anti-c5′N1A Antibodies 

Originally, IBM was considered to be a cytotoxic T cell-mediated disease with no humoral autoimmunity, until the finding of immunoglobulin transcripts in IBM muscle samples, and the finding of a 43-kD autoantibody, identified as being directed to cytosolic 5-nucleotidase 1A (anti-NT5c1A or anti-cN1A) [9,103,104]. About half of IBM patients (33–76%) are positive for this biomarker [71]. Anti-cN1A targets multiple cN-1A epitopes [105]. However, the anti-cN1A antibodies were found in other autoimmune diseases, such as SjS, systemic lupus erythematosus (SLE), juvenile dermatomyositis, and others, and even in healthy controls [71,104,105].

The cytosolic 5′nucleotidases (NT5cs) play a central role in the regulation of the purine nucleotide pool.

An enzyme, cN-1A, is involved in the hydrolysis of adenosine monophosphate to adenosine. It is also involved in the dephosphorylation of nucleotides to nucleosides [105]. Furthermore, cN1A is highly expressed in skeletal muscles, and is involved in metabolic regulation, energy balance, and cell replication [73].

Clinically, the presence of anti-cN1A antibodies in IBM patients was associated with muscle weakness severity, with dysphagia and a higher adjusted mortality risk [72,104]. Additionally, anti-cN1A-positive patients had more cytochrome oxidase deficient fibers than the negative patients, thus, reflecting the involvement of these antibodies in the occurrence of mitochondrial abnormalities in IBM [72]. The expression of cN1A is higher near the vacuoles, and may reflect specific mechanisms of IBM, such as myonuclear degeneration [7]. Moreover, passive immunization in IBM models with anti-cN1A-positive IgG resulted in intracytoplasmic aggregation of the anti-apoptotic molecule p62/SQSTM1 in myofibers, associated with macrophage accumulation [73]. The anti-cN1A autoantibodies were associated with reduced muscle cN1A expression [73].

Serum cN-1A activity was decreased in breast cancer, correlated with muscle damage parameters, likely related to anti-cN1A autoantibodies [74]. Therefore, it is tempting to presume that muscular cN-1a could become autoantigenic to the breast cancer-associated paraneoplastic myositis [106].

The knockdown of cN1A increases the phosphorylation of the AMP-activated protein kinase (AMPK), a regulator of mitochondrial dynamics and biogenesis [74,107]. This AMPK increases mitochondrial mass and activity [76]. When AMPK is activated in low cellular energy states, it turns on the catabolic pathways, producing ATP, and turns off the ATP-requiring anabolic pathways [74,77]. Therefore, AMPK activation inhibits mTORC1 (the mechanistic target of rapamycin complex-1), and thus, contributes to muscle wasting by reducing protein synthesis [64]. Moreover, AMPK activation results in upregulation of the muscle-specific ubiquitin ligases muscle atrophy F-box and muscle RING finger 1, which are markers of muscle catabolism [73]. Therefore, due to this cross-talk, anti-cN1A antibodies may affect protein degradation in muscle [73].

Besides metabolic regulation, AMPK may be involved in the functions of the tumor-suppression kinase LKB1, an upstream kinase activating AMPK [77]. Generally seen as a tumor suppressor in early lesions, AMPK promotes cancer in the advanced stages by protecting the neoplastic cells against metabolic stress, mainly when the tumor nutrient supply becomes insufficient [77]. Therefore, the relationship between the cN1A antibodies and tumors is complex and depends on the disease stage.

## 5. Mitochondrial Abnormalities 

Mitochondrial abnormalities in IBM are also related to autoimmunity, as in DM [4,108]. Thus, IBM can be considered an “acquired mitochondrial disease with extras” [78]. In IBM, mitochondrial abnormalities with reduced mitochondrial size, dynamics, and ATP production defects are frequent (Figure 1) [78,79]. Respiratory-deficient fibers at different stages of mitochondrial dysfunction are seen in IBM, with a strong correlation between the severity of inflammation and the degree of mitochondrial changes and atrophy [6]. The mitochondrial dysfunction in IBM is progressive, and the clonal expansion and mtDNA rearrangements are likely secondary to inflammation, possibly to a viral infection, either causing CD8+ T cells accumulation or MHC I upregulation with an ER stress response and consecutive mitochondrial alterations [6].

Mitochondria also influence all steps of oncogenesis, from malignant transformation to tumor progression and response to therapy [80]. Cancer cells are resistant to apoptosis, and mitochondrial adaptations may favor cancer cell survival [80,81]. Moreover, some mitochondrial metabolites may be oncogenic by themselves [80].

The growth differentiation factor GDF15, also called macrophage inhibitory cytokine 1, is a marker of mitochondrial disease, and is increased in both IBM and in cancers [78,79,84]. The GDF15 factor is upregulated by proinflammatory cytokines in stress, inflammation, or aging, and induces a lean phenotype for survival [109,110]. Additionally, GDF15 is elevated in various types of cancers, most prominently in prostate, urothelial, renal, melanoma, and colorectal cancers [110].

Sirtuins, a family of proteins associated with longevity and cell survival, are important in energy metabolism [111]. The sirtuin-1 (SIRT1) directly regulates mitochondrial function and response to ischemic stress [81]. Additionally, SIRT1, activated by AMPK agonists and others, catalyzes the nicotinamide adenine dinucleotide (NAD) deacetylation [64,81,111]. In IBM, SIRT1 is low in muscle fibers, despite the increased SIRT-1 mRNA and protein, and decreased SIRT-1 may contribute to NFkB activation in IBM [82]. Of interest, in an experimental model of IBM, sirtuin-3 signaling was activated by resistance exercise, resulting in an improvement of mitochondrial quality control, dynamics, and oxidative capacity [82,83].

In cancer, SIRT1 may have dual functions as a tumor promoter or tumor suppressor. Indeed, SIRT1 directly influences tumor progression, metastasis, resistance to apoptosis, autophagy, DNA repair, and other oncogenic mechanisms [81,111]. Downregulation of SIRT1 in T cell leukemia/lymphoma enhances apoptosis, cell cycle arrest, and sensitivity to chemotherapy [111].

Additionally, SIRT1 negatively regulates p53-induced cellular senescence [112]. The p53 molecule, the genome guardian, and one of the most important tumor suppressors, is frequently mutated in solid tumors [112], and is also a hub gene in IBM [33].

The metabolic profiles of cancer cell and activated cytotoxic CD8+ T cells are notably similar, promoting their survival and proliferation in a hostile microenvironment, with increased ATP requirements [70]. Both T cells and cancer cells suppress their mitochondrial oxidation and fatty acid uptake, and rely upon mitochondrial fatty acid oxidation [70].

## 6. Autophagy in IBM and Cancers 

IBM is a degenerative muscle disease associated with intra-muscle fiber multi-protein aggregates, proteasome inhibition, ER stress, and decreased lysosomal degradation [82]. Aggregation of proteins and inclusions in the myofibers can result from exposure to inflammatory mediators, such as IFNγ and IL-1β [4]. The IBM vacuoles contain intermediate filaments, extracellular matrix proteins, and the proteins involved in cell stress response, protein quality control, and protein degradation, among others [89]. The multi-protein congophilic aggregates in vacuoles include beta-amyloid, amyloid precursor protein, alpha-beta crystalline, phosphorylated tau, alpha-synuclein, dysferlin, myostatin, heat shock protein 70, and others [3,9,11]. Nevertheless, the new histochemical markers p62 (SQSTM1), LC3, and TDP-43, involved in autophagy, unfolded protein response (UPR) and ER stress, could probably reflect better the degenerative changes in IBM [59,89].

Autophagy, a major degradative pathway for cytosol and organelle turnover, is critical for degrading potentially cytotoxic or damaging proteins and damaged mitochondria, increasing cell resilience. Autophagy is crucial in T cell development, survival, and proliferation [113]. In tumors, defective autophagy can result in cell damage via the accumulation of deleterious constituents, whereas its overactivation can increase tumor survival by providing fuel to cancerous cells [81].

Sarcoplasmic aggregates of the autophagy-associated proteins p62 (encoded by the sequestosome 1 gene (SQSTM1) and LC3 are a pathologic hallmark of IBM [73]. Moreover, anti-cN1A antibodies induce P62 aggregation in cell cultures and mouse experimental myositis [4,73]. Belonging to the sequestosome that functions as a storage place for ubiquitinated proteins, P62 is an autophagy adaptor protein interacting with the autophagosomal membrane protein LC3 (microtubule-associated protein 1 light chain 3) [86,113]. Additionally, p62 intervenes on multiple metabolic pathways in oxidative stress, inflammation, neurodegeneration, and cancer [86]. The IBM vacuoles proteomics also identified missense variants of FYVE and coiled-coil domain-containing protein-1 (FYCO1), an LC3-binding protein, which links autophagic and endocytic pathways [89].

Autophagy adaptors, such as p62, function as tumor suppressors, and p62 accumulation through autophagy defects is found in many tumors, such as digestive, lung, breast, prostate, and gynecological cancers, as well as in melanoma and others [86,87]. Moreover, p62 contributes to apoptosis regulation through the activation of polyubiquitinated caspase-8 [88]. Additionally, LC3, associated with autophagosome formation, is a marker of poor tumor differentiation in cancer cells [88]. In cancers, such as squamous cell carcinoma, FYCO1 mediates microtubule-dependent autophagosome transport and maturation, and regulates post-mitotic midbodies degradation [90]. The accumulation of post-mitotic midbodies in cancer cells is involved in increased invasiveness and metastatic potential [90].

The TDP-43 (TAR-DNA-binding protein-43) protein is a member of the hn-RNP family, acting as a RNA and DNA binding regulator, mediating RNA metabolism and transcription regulation [114]. In IBM, pathological TDP-43 accumulates with mitochondria in degenerating muscle fibers with toxic effects [91]. These TDP-43 signaling alterations have been associated with cancers, either as a tumor promoter (upregulated in breast, lung, hepatocellular cancer, or glioblastoma), or as tumor suppressor (downregulated in cervical cancer, colon, neuroblastoma, and others) [114]. The precise molecules mediated by TDP-43 in various settings and its therapeutic consequences are yet unclear [114].

Other proteins, such as β-amyloid, amyloid precursor protein, hyperphosphorylated tau, and others, accumulate in IBM [4]. In IBM, β-amyloid expression, along with IFNγ, induces myotubes or myoblast apoptosis [115]. The homologues of amyloid precursor protein APLP1 and APLP2 are overexpressed in many cancers [116]. Nevertheless, amyloid Aβ inhibits the growth of some human cancer cells, triggering autophagy in glioma and neuroblastoma cell lines [117].

Tau is a microtubule-associated protein which promotes microtubule assembly [118]. Hyperphosphorylated tau encountered in IBM functions deficiently, as its affinity for microtubules is reduced [118]. Tau is a prognostic marker for cancer and is involved in resistance to therapy in various tumors [119].

Other proteins included in the rimmed vacuoles in IBM, identified through proteomic analysis, include chaperones, part of the intracellular network controlling biosynthesis and the correct folding of proteins and degradation of misfolded or defective proteins [85]. Chaperones interact with autophagic pathways and the ubiquitin proteasome system (UPS) [85]. Chaperonines, a subclass of chaperones, including the mitochondrial heat shock protein 60, the T-complex protein-1 (TCP-1) subunits, and others, temporarily encapsulate the proteins for their proper folding [85]. Additionally, TCP-1 is overrepresented in IBM vacuoles early in the disease [85]. Intriguingly, chaperonin-containing TCP-1 promotes tumor progression, chemoresistance, and metastasis, and its inhibition could overcome cancer resistance to therapy [92].

## 7. Cell Cycle Abnormalities in IBM and Cancers

### 7.1. The Ubiquitin-Proteasome System in IBM and Cancers

The IBM disorder is characterized by muscle atrophy, which occurs when protein degradation exceeds their synthesis [120]. The UPS is recognized as a major intracellular protein degradation system, important for muscle homeostasis, but UPS is also involved in myoregeneration [93]. Excessive proteolysis in skeletal muscle leads to muscle atrophy, while the inhibition of proteolytic pathways is also associated with muscle wasting and weakness [93].

Most proteins undergo degradation after they are attached to a multi-ubiquitinated chain and targeted by the 26S proteasome, which initiates an ATP-dependent degradation process [93]. The muscle UPS activity decreases with age [93]. In IBM, the defective or inhibited elimination of ubiquitinated misfolded or unfolded proteins results in the accumulation of the proteic aggregates in the myofibers [93]. A mutant ubiquitin B (UBB1+) inhibits the proteasome, thus, contributing to the accumulation of misfolded proteins, namely amyloid β and phosphorylated tau, in IBM [121].

Ubiquitin ligases related with UPS in skeletal muscle have been recently reviewed [93]. The ubiquitin ligase muscle atrophy F-box (Atrogin-1/MAFbx) is a skeletal and cardiac muscle. The specific F-box contains protein and is activated in cachexia, including tumoral cachexia; its expression precedes muscle loss and is increased in IBM [94]. The expression of ubiquitin ligases is regulated by a transcription factor, Forkhead box O (FoxO), which at its turn is negatively regulated by the insulin-Akt pathway, and activated by proinflammatory cytokines, leading to atrophy [120].

There is crosstalk with autophagy system in muscle-specific proteasome dysfunction [93]. Ubiquitinated proteins are degraded by autophagy, and autophagy is enhanced in UPS dysfunctions [93]. The highly conserved ubiquitin pathway is a crucial regulator of cancer, and its alterations disturb signaling pathways, including cell cycle progression and DNA damage repair [95].

### 7.2. Cell Cycle in IBM and Cancers

Some of the same oxidative stress and UPS abnormalities affect IBM and can regulate the cellular cycle [96]. Cell cycle reentry involves mature muscle cells, and satellite cells are not activated in IBM [96]. When not resulting in mitosis, cell cycle reentry is likely to initiate apoptosis [96]. Induction of caspases and heat shock proteins, which regulate cell cycle at different phases, was observed in IBM [96]. Increased expression of cell cycle markers Ki67, PCNA, and cyclins D1 and E was also noted in IBM, but also in polymyositis, as compared to controls [96]. The proliferation marker Ki67 is strongly associated with tumor growth [97]. Cyclins are normal cell cycle control proteins; cyclins D1 and cyclin E, associated with high chromosomal instability, are overexpressed in several tumors, including in head and neck cancers [98]. Inflammation and β-amyloid may independently activate cell cycle reentry, and in IBM both degenerative (amyloid) and inflammatory (cellular) features are responsible for stimulating myofibers to cross through the G/S1 phases and re-enter the cell cycle [96].

### 7.3. MicroRNAs in IBM

MicroRNAs (miRNAs) are short, non-coding single-stranded RNA targeting certain mRNAs for translational suppression and/or activation [57]. The particular miRNA in muscle diseases, called myomiRs, are dysregulated in IIMs [122]. For instance, in IBM, miR-133, acting on mitochondria, is markedly reduced [78]; miR-133 is also a tumor suppressor, and is decreased in acute myeloid leukemia and in other cancers [99].

## 8. Other Mechanisms 

Calcium is a universal secondary messenger in muscle, regulating contraction, apoptosis, and other processes, but it also plays a role in T cells, controlling their proliferation, differentiation, function, and fate [100,101]. Furthermore, Ca^2+^ homeostasis dysregulation is involved in the defective cytotoxic CD8+ T cells apoptosis in IBM [101]. The genes regulating Ca^2+^-induced T cell apoptosis and other Ca^2+^-dependent molecules involved in cell survival and inflammation, such as the NR4A family genes and the T cell specific protein tyrosine kinase LCK, are significantly dysregulated in IBM muscles [101]. Moreover, Ca^2+^ dyshomeostasis is closely linked to mitochondrial defects [100]. Calpains, namely calcium-activated cysteine proteases, are involved in various cellular processes, including signal transduction, survival, proliferation, apoptosis, migration, and invasion [102]. In IBM, cytosolic Ca^2+^ elevations enhance the calpain-1 activation, which further activates other Ca^2+^-regulatory proteins, leading to excessive intracellular Ca^2+^ [100]. Therefore, a functional calpainopathy has been proposed in the IBM pathogenesis [100]. Aberrant calpain activity is also involved in various cancers, generally negatively associated with the outcome, and, thus, calpain inhibition is a new therapeutic strategy in oncology [102].

## 9. Therapies in IBM and Cancers

An efficient therapy for IBM is a currently unmet need. The resistance to therapy in IBM may be related to the more aggressive and neoplastic nature of T-invasive clones, which are long-lived, resistant to apoptosis, and difficult to eradicate [28]. Elimination of senescent T cells (with agents called senolytics) may be obtained by targeting apoptosis, chaperones, or histone modifications [47]. The IFNγ upregulation in IBM is a strong argument for a potential effect of JAK inhibitors in IBM [54]. Pan-JAK inhibitors deplete the senescent cells in aged mice, alleviating SASP and frailty (although they may reactivate latent viruses resulting in shingles) [47]. Inhibition of STAT3 could prove useful for IBM and LGL, as well as for cancer-induced cachexia [63,64,65,123]. Calcitriol, the active form of vitamin D, decreases STAT1 and STAT3 phosphorylation with potential effectiveness in T-LGLL [124]. Calcitriol directly acts on malignant cells and on activated T cells, decreasing inflammation and promoting anti-inflammatory cytokines production [124].

Blocking PD-1, although important in many cancers, is associated with immune phenomena. Interesting, while senescence is involved in preventing malignancies, exhaustion may reduce the risk of excessive immune activation [45]. Therefore, a long-time reversal of T cell senescence could increase cancer risk, while PD-1 inhibition blocking exhaustion risks immune activation [45]. Selectively targeting downstream components of exhaustion and senescence pathways would probably pave the way to T-tailored therapies in personalized medicine [45]. Better understanding of pathogenesis, the difference from other types of myositis, as well as patients’ subclassification could help in predicting the response to a certain therapy, in order to tailor the treatment [125].

## 10. Concluding Remarks and Future Perspectives

The terminally differentiated, CD8+ highly cytotoxic T cells expressing NK features are central in the pathogenesis of IBM. Nevertheless, despite the phenotypic changes, mostly aiming to fight against tumors, the immunosenescent CD8+CD28 null T cells are paradoxically involved in many cancers as well. Indeed, IFNγ may play a central role in IBM, mostly during the early stages of the disease [54]. There are intermingled subcellular mechanisms in IBM and cancer, and probably their co-existence is underestimated.

In the assessment of IBM patients, a high index of suspicion, manual reading of blood smears to reveal an increased population of LGL, along with cytopenia, lymphocytosis, and flow cytometry are necessary to detect a LGL clonal proliferation [28]. Besides LGL, there is also a need to detect and report all cancers in this aging population where the cancer risk is high, as there is a direct biological link between the aged phenotype and cancer risk [126]. In IBM, the cancer frequency distribution was as in the similar age population, with the most common cancers being prostate, colorectal, and hematologic [12,126]. Additionally, most panels for myositis-specific and myositis-associated autoantibodies do not include cN1A to recall an IBM, despite their low specificity. After finding a cancer-associated myositis, the disease is not always explored further from muscular point of view, and possibly cancer-associated IBM are overlooked, inasmuch as muscle atrophy and dysfunction could be attributed to cancer itself. In clinical practice, disease-specific quality indicators and outcome measures are recommended for the assessment [127]. Besides other therapies, physical rehabilitation improves function, and may also increase mitochondrial quality control [83].

The increasingly identified role of T cell subpopulations in cancers and in tumor niches could bring about valuable insights into IBM pathogenesis and, hopefully, its therapy. Additionally, the crosstalk between metabolic and mitogenic pathways, partly mediated by AMPK in cancers, could be shared by IBM. The potential links between IBM and cancers deserve further interest, in order to increase our understanding of the pathogenesis, to search for efficient therapies in IBM, and to improve muscle function, life quality, and survival in both diseases.

## Figures and Tables

**Table 1:** Dysfunctional cytotoxic CD8 T cell markers [47,48,49].

Characteristics	Exhausted T Cell	Senescent T Cell
Cell cycle	Reversible block	Irreversible block
T cell markers	CD44+/−	Loss of CD27, CD28, +/−CCR7 Re-expression of CD45RA
NK markers	-	CD57++, KLRG1++
Metabolic	PI3k/AKT/mTOR+/− MAPK++	PI3k/AKT/mTOR+/−
Expression	PD-1, TIM1, LAG3, CTLA4, TIGIT	perforin, IFNγ, TNFα, granzyme B, KLRG1
Function	Defective effector functions	Effector functions (SASP)

Legend: KLRG1—Killer Ig-like receptors, effector memory (CD27-CD45RA); SASP—senescent cell secretory phenotype; CD45—tyrosine phosphatase family regulating T cell and B cell receptor signaling; TIGIT—T cell immunoreceptor with immunoglobulin and ITIM domain; TIM-1—T cell immunoglobulin and mucin domain-containing protein 1.
